# Neurotoxic Doses of Chronic Methamphetamine Trigger Retrotransposition of the Identifier Element in Rat Dorsal Dentate Gyrus

**DOI:** 10.3390/genes8030096

**Published:** 2017-03-06

**Authors:** Anna Moszczynska, Kyle J. Burghardt, Dongyue Yu

**Affiliations:** 1 Department of Pharmaceutical Sciences, Eugene Applebaum College of Pharmacy and Health Sciences, Wayne State University, Detroit, MI 48201, USA; dongyue.yu@wayne.edu; 2 Department of Pharmacy Practice, Eugene Applebaum College of Pharmacy and Health Sciences, Wayne State University, Detroit, MI 48201, USA; kburg@wayne.edu

**Keywords:** methamphetamine, DNA methylation, identifier element, short interspersed elements, rat brain, dentate gyrus, retrotransposition

## Abstract

Short interspersed elements (SINEs) are typically silenced by DNA hypermethylation in somatic cells, but can retrotranspose in proliferating cells during adult neurogenesis. Hypomethylation caused by disease pathology or genotoxic stress leads to genomic instability of SINEs. The goal of the present investigation was to determine whether neurotoxic doses of binge or chronic methamphetamine (METH) trigger retrotransposition of the identifier (ID) element, a member of the rat SINE family, in the dentate gyrus genomic DNA. Adult male Sprague-Dawley rats were treated with saline or high doses of binge or chronic METH and sacrificed at three different time points thereafter. DNA methylation analysis, immunohistochemistry and next-generation sequencing (NGS) were performed on the dorsal dentate gyrus samples. Binge METH triggered hypomethylation, while chronic METH triggered hypermethylation of the CpG-2 site. Both METH regimens were associated with increased intensities in poly(A)-binding protein 1 (PABP1, a SINE regulatory protein)-like immunohistochemical staining in the dentate gyrus. The amplification of several ID element sequences was significantly higher in the chronic METH group than in the control group a week after METH, and they mapped to genes coding for proteins regulating cell growth and proliferation, transcription, protein function as well as for a variety of transporters. The results suggest that chronic METH induces ID element retrotransposition in the dorsal dentate gyrus and may affect hippocampal neurogenesis.

## 1. Introduction

Transposable elements are non-coding pieces of DNA with the ability to “jump” to different locations in the genome upon activation [1]. They can control genes epigenetically when inserted (transposed) into genes or gene regulatory regions. Activation of transposable elements most often depends on DNA hypomethylation at promoter regions [2,3,4]. Their transposition to another location in the genome takes place during both embryonic and adult neurogenesis [1,5,6,7]. Short interspersed elements (SINEs) are non-autonomous transposable elements known as short retrotransposons [8]. They are short DNA sequences with a length of 80–400 bp that are dispersed over the eukaryotic genome, are amplified by a reverse transcription and retrotranspose by a copy-and-paste mechanism [9]. In contrast to long retrotransposons (LINEs), SINEs do not code for a reverse transcriptase [4,10] and, therefore, they must interact with endogenous cellular factors to retrotranspose. Specifically, SINEs are reverse-transcribed by partner LINE-1s [10,11,12] and interact with cellular proteins, including poly(A)-binding proteins (PABPs) to form RNA-protein (RNP) complexes in the cytoplasm [13] that later translocate to the nucleus for the insertion of SINEs into DNA.

Identifier (ID) elements belong to the group of simple SINEs and are widespread in the rat genome [14]. The human counterpart to the ID element is the *Alu* element, while its mouse counterparts are the B1 and B2 elements. ID elements are about 100 bp long and consists of a core domain containing an internal RNA polymerase III promoter, a poly(A) region and 5′- and 3′-flanking regions [14,15]. Due to their widespread presence in the rat genome, ID elements have been used as determinants of global methylation in rat tissues [16]. Human and mouse counterparts of the rat ID element were demonstrated to be activated by hypomethylation [17]; therefore, ID element transcription is most likely activated by removal of methyl groups within its sequence. Exposure to heat shock, genotoxic agents, mechanical damage or ischemia increases SINEs transcription, which can lead to their retrotransposition [10,11,18,19,20,21]. Increases in SINE transcripts, as well as SINE retrotransposition can regulate gene expression, with the former having a short-term and the latter a long-term effect. To date, no studies have examined ID element methylation status or its retrotransposition after neurotoxic doses of methamphetamine (METH).

METH is a widely-abused central nervous system (CNS) psychostimulant, which reduces hippocampal volume and induces apoptosis in the hippocampus in experimental animals and humans, particularly when administered at high doses [22,23,24,25,26]. METH also affects adult neurogenesis in the hippocampus [22,27,28]. These molecular events are thought to underlie a variety of cognitive impairments observed in chronic human METH users [29]. Relatively little is known about epigenetic changes induced by neurotoxic doses of METH in adult hippocampus. To our knowledge, only our laboratory investigated METH effects on transposable elements in the hippocampus and found increased LINE-1 expression in the dentate gyrus [30]. The aim of the present investigation was to determine whether binge or chronic administration of neurotoxic METH doses leads to retrotransposition of the ID element in the dentate gyrus of adult male rats. Toward this goal, we measured ID element methylation over time, as well as the diversity in amplification of the element in DNA samples from the dentate gyrus of saline- and METH-treated rats. We also assessed the levels of PABP1, a putative ID element-binding protein with a high affinity for the poly(A) tail of mRNA [31] and regulator of SINE retrotransposition [13].

We have found that binge METH causes hypomethylation of CpG-2 site within the ID element sequence at 1 h and 24 h after the last injection of the drug, while chronic METH causes hypermethylation of this site at 1 h after the last METH injection as compared to saline-treated control rats. The methylation status of the ID element returned to basal levels by the seventh day after binge METH and by 24 h after chronic METH. Seven days after chronic METH, the CpG-2 site displayed small, but statistically significant hypermethylation. An increase in the levels of PABP1 was detected at 24 h after binge METH and after two days of chronic METH administration. Regarding METH-induced ID element retrotransposition, the loci with the greatest difference in relative ID element amplification between METH and control samples were mapped to genes encoding for proteins regulating cell growth and proliferation, transcription, protein function as well as for a variety of transporters.

## 2. Materials and Methods

### 2.1. Animals

Adult male Sprague-Dawley rats (Harlan, Indianapolis, IN, USA) (weighing 250–300 g on arrival) were pair-housed under a 12-h light/dark cycle in a temperature- (20–22 °C) and humidity-controlled room. Food and water were available *ad libitum*. The animals were allowed to acclimate for a week before the start of the study. All animal procedures were conducted between 7:00 a.m. and 7:00 p.m. in strict accordance with the National Institutes of Health (NIH) Guide for Care and Use of Laboratory Animals and approved by the Institutional Animal Care and Use Committee (IACUC) at Wayne State University (Animal Protocol #A 05-07-13). The description of the animal procedures meets the Animal Research: Reporting of in Vivo Experiments (ARRIVE) recommended guidelines described by The National Centre for the Replacement, Refinement and Reduction of Animals in Research [32].

### 2.2. Administration of Methamphetamine

**Figure 1:**
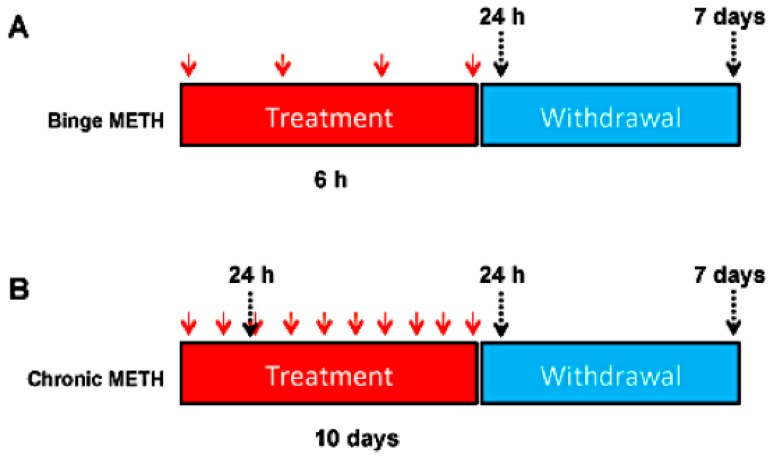
Experimental design. Adult male Sprague-Dawley rats were administered saline (1 mL/kg), binge METH (4 × 10 mg/kg, i.p. every 2 h) (**A**) or chronic METH (20 mg/kg/day for 10 days, i.p.) (**B**). Core body temperatures (°C) were measured before treatments and 1 h after each METH or saline injection. The rats were sacrificed 24 h or 7 days after the last dose of saline or METH or on the 3rd day of chronic METH regimen (24 h after the 2nd dose). The red arrows indicate the injection times, whereas the black arrows indicate sacrifice times.

(+)-Methamphetamine hydrochloride (METH) (Sigma-Aldrich, St. Louis, MO, USA) or saline was administered to rats in a binge (10 mg/kg, every 2 h in four successive intraperitoneal (i.p.) injections) or chronically (20 mg/kg, daily, for 10 days, i.p.). Both paradigms are established as models of METH neurotoxicity in rats and other experimental animals. METH neurotoxicity (neurodegeneration or damage to neuronal components within neuronal terminals or cell bodies causing dysregulation of neuronal function) is associated with hyperthermia [33], which peaks at approximately at 1 h after i.p. injection of METH. Therefore, core body temperatures were measured via a rectal probe (Thermalert TH-8; Physitemp Instruments, Clifton, NJ, USA) before the treatments (baseline temperatures) and 1 h after each METH or saline injection. Rats were sacrificed by decapitation at 1 h, 24 h or 7 days after the last injection of the drug or saline. For some analyses, rats were sacrificed 24 h after the first two days of chronic METH administration, at which point the rats were injected with the same total dose of METH as the binge METH rats (2 × 20 mg/kg and 4 × 10 mg/kg, respectively). The experimental design is shown in Figure 1.

### 2.3. Tissue Collection

The brains were removed and dissected out into discrete brain areas (striatum, dentate gyrus, Ammon’s horn, prefrontal cortex and cerebellum) and stored at −80 °C until assayed. The subgranular zone (SGZ) was dissected out together with the dentate gyrus. The dentate gyrus was dissected out according to a previously described protocol [34] modified for the rat brain. Briefly, the brain was cut sagittally to divide the hemispheres, which were then placed medial side up after removal of the regions posterior to lambda. The dentate gyrus and Ammon’s horn (CA1, CA2 and CA3) (which were visible upon removal of thalamus and hypothalamus) were dissected out using fine tip surgical instruments. Muscle tissues (negative control for METH effects) were also collected and stored at −80 °C until analyzed.

### 2.4. Polymerase Chain Reaction and Pyrosequencing

**Figure 2:**
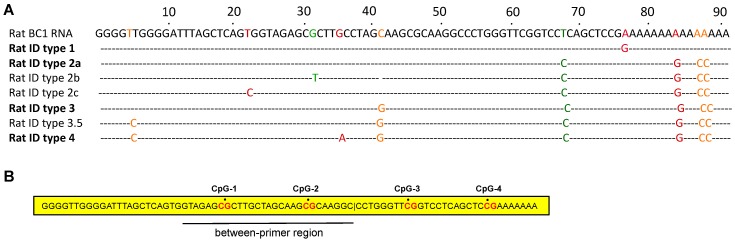
Schematic illustration of ID element sequences in the rat. (**A**) Alignment of seven consensus sequences of rat ID subfamilies with the rat BC1 RNA gene. The dot indicates the base identity at each position. The lower case letters indicate the ambiguity at that position. Each subfamily is named in sequential order, types 1–4, based on the age of each subfamily. Three major subfamilies are in bold type. The diagnostic changes of these major subfamilies and one minor subfamily, type 3.5, accumulate progressively from older to younger subfamilies (bold-type). The two minor subfamilies, types 2b and 2c, are derived from the previous major subfamily, type 2 [14]; (**B**) Rat BC1 RNA with CpG sites and the between-primers region.

Each ID element core contains four CpG nucleotides (Figure 2). DNA methylation of the first two CpG sites within the ID element sequence was determined by pyrosequencing at EpigenDx Inc. (Hopkinton, MA, USA), using glyceraldehyde-3-phosphate dehydrogenase (GADPH) as a reference gene [35]. Briefly, 500 ng of DNA from each rat brain tissue sample were first treated with bisulfite and subsequently purified using Zymo Research DNA columns (Zymo Research, Irvine, CA, USA). A 1/20th eluted solution was used for each PCR. Biotinylated PCR products were bound to Streptavidin Sepharose HP (GE Healthcare, Waukesha, WI, USA). Immobilized PCR products were purified using Pyrosequencing Vacuum Prep Tool (Qiagen, Valencia, CA, USA) according to the manufacturer’s instructions. The 0.2 µM pyrosequencing primer was annealed to purified single-strained PCR product. The PCR products (10 uL) were sequenced using the Pyrosequencing PSQ96 HS System (Biotage AB, Charlotte, NC, USA). The methylation status of each locus was analyzed individually as a T/C SNP using QCpG software (Pyrosequencing, Qiagen). The analysis was performed on duplicate samples, and the samples were averaged. The data are expressed as percent (%) of methylation (mean ± SEM).

### 2.5. Immunohistochemistry

Brain tissue from the rats sacrificed by decapitation at 24 h after the last injection of METH or saline was fixed in 4% paraformaldehyde for 24 h, then incubated in 20% and 30% buffered glycerol concentrations for 24 h each (4°C). Every other of the coronal sections (20 μm, 3 sections/rat) from the dentate gyrus (−3.12–−4.68 mm from Bregma) was examined for the levels of PABP1, using the immunofluorescence technique. Sections were first pretreated with 1× citrate buffer for 40 min at 70 °C, then allowed to cool to room temperature before being blocked in blocking buffer (phosphate-buffered saline (PBS) pH 7.4, 0.1% Triton X-100, and 5% bovine serum albumin (BSA)) for 1 h at room temperature. The sections were then incubated overnight at 4 °C with a rabbit anti-PABP1 primary antibody (1:100, #4992, Cell Signaling Technology, Danvers, MA, USA). The next day, the sections were incubated for 3 h at room temperature with anti-rabbit Alexa-594 secondary antibody (1:2000, Life Technologies, Carlsbad, CA, USA). The incubations with primary and secondary antibodies were separated by three 5 min-long washes with PBS that contained 0.1% Triton and 5% BSA. The nuclei were labeled with DRAQ5 dye (Life Technologies). The sections were mounted on slides using Flouromount mounting medium (Sigma-Aldrich). Images (taken along the dentate gyrus) were captured using a Leica TCS SPE-II confocal microscope under the 63 × oil objective (Leica, Buffalo Grove, IL, USA United States. PABP1 immunofluorescence, measured in 3 non-overlapping areas on each slice, was first averaged per slice and then averaged per rat before statistical comparison of the controls and METH groups. The data are expressed as PABP1 immunofluorescence normalized to saline controls (mean ± SEM).

### 2.6. Next-Generation Sequencing and Sequence Diversity Analysis

Analysis of dynamic transposable element activity via next-generation sequencing (NGS) technologies has been difficult in the past because it is challenging to align sequencing reads to repetitive regions of the genome [36]. Computational approaches to profiling the sequence diversity of ID elements have since been introduced to help overcome these barriers, and our group has applied these strategies for detection of ID elements. This method first characterizes the dynamic ID element activity by quantifying the relative proportion of clonal population subtypes, using the BC1 consensus sequence as a reference, within this ID element family, followed by mapping of the most significant subtypes back to their genomic locations [15]. This approach has an advantage over the traditional alignment approach because it considers all ID elements in a population and is agnostic to mapping, whereas the traditional approach must focus on the rare subset of sequences that mapped uniquely to the reference genome. We were able to estimate the change in total copy number associated with ID element activity in METH-treated rats relative to controls. Briefly, dorsal dentate gyrus samples were sequenced using Ion Torrent Sequencing technology after amplification of the target BC1 ID element master gene (BC1 reference sequence: GGTTGGGGATTTAGCTCAGTGGTAGAGCGCTTGCCTAGCAAGCGCAAGGCCCTGGGTTCGGTCCTCA). In order to describe the diversity of the aligned ID elements after sequencing, a set of mutations, or discrepancies from the reference, were determined for each read, and the total number of reads was determined for each unique ID element subtype. To avoid intrinsic error associated with sequencing, subtypes with 100 or more normalized per million reads were then grouped according to treatment condition, and the ID element subtypes with the greatest change relative to controls were identified. The top sequences (defined by a *p*-value <0.05) from the identified subtypes were aligned to a reference genome (Rat Genome Sequencing Consortium 6.0/ Rattus norvegicus 6 (RGSC 6.0/rn6) using the Bowtie2 read aligner tool [37]. The number of times each sequence aligned to the genome and its location in the genome was also determined, and only genomic alignments with zero mismatches were kept for interpretation. ID subtypes that did not uniquely match at least once to the reference genome were not included in the results table.

### 2.7. Statistical Analysis

All data were tested with the Levene test for the heterogeneity of variances and with the Shapiro-Wilk test for normality. Two-way repeated-measures ANOVA followed by the Student-Newman-Keuls post hoc test was performed on the temperature data. Two-way ANOVA followed by the Fisher LSD post hoc test was performed on the methylation data. As our sample sizes were small and group comparisons were preplanned, the Fisher LSD test was chosen as a post hoc test to avoid making the type II error (accepting a null hypothesis that is actually false), as the Student-Newman-Keuls or Tukey’s HSD test may be too conservative for this study. This strategy will maximize our ability to detect differences in a small, pilot sample, and so, findings from using this strategy will likely need to be replicated in further samples in the future. Student’s unpaired two-tailed *t*-test was performed on the immunochemistry data. The NGS data were analyzed by Student *t*-tests comparing the number of reads in ID element subtypes between METH- and saline-treated rats. The results are expressed as the mean ± SEM. Statistical significance was set at *p* < 0.05.

## 3. Results

### 3.1. The ID Element Is Similarly Methylated in Different Rat Brain Regions

**Figure 3:**
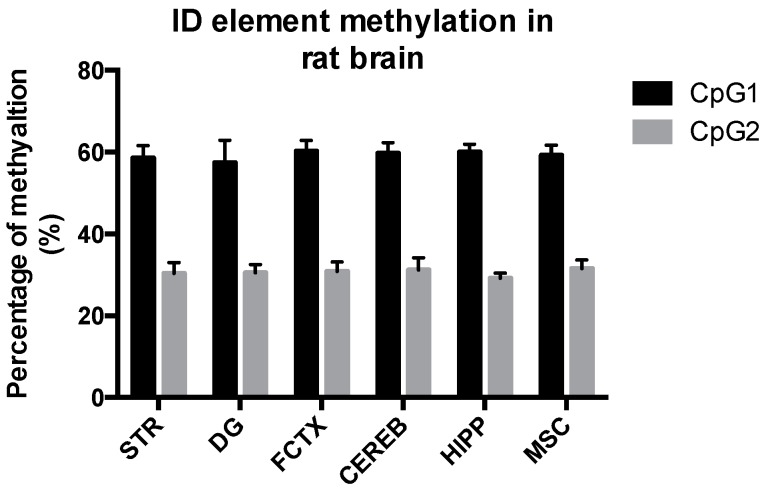
Methylation status of ID element CpG-1 and CpG-2 sites in several brain areas and muscle tissue in the rat. The methylation percentage of CpG-1 and CpG-2 in five different brain areas, namely the striatum, dentate gyrus, Ammon’s horn, frontal cortex and cerebellum, as well as in muscle tissue of the rat, was similar: 57%–60% at CpG-1 and 29%–31% at CpG-2. The data are expressed as the mean ± SEM (*n* = 12). Abbreviations: CEREB, cerebellum; CpG, C-phosphate-G; DG, dentate gyrus; FCTX, frontal cortex; HIPP, CA1-CA3 of the hippocampus (Ammon’s horn); MSC, muscle, STR, striatum.

To our knowledge, the methylation status of ID element CpG sites in different brain areas has not been determined. To assess whether there are regional difference in methylation status of the ID element, we assessed methylation of two CpG sites, CpG-1 and CpG-2, in five different brain areas, namely the striatum, dentate gyrus, Ammon’s horn, frontal cortex and cerebellum, as well as in muscle tissue of the rat. The methylation percentages for each rat brain area were very similar, 57%–60% at CpG-1 and 29%–31% at CpG-2. Muscle tissue displayed similar methylation status as the brain. The data are summarized in Figure 3.

### 3.2. Binge and Chronic High-Dose METH-Induced Hyperthermia in the Rat

**Figure 4:**
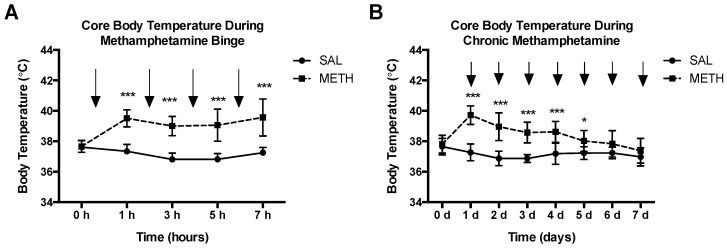
METH-induced hyperthermia. Adult male Sprague-Dawley rats were administered saline (1 mL/kg), binge METH (4 × 10 mg/kg, i.p. every 2 h) or chronic METH (20 mg/kg/day for 10 days, i.p.). Core body temperatures (°C) were measured before treatments and 1 h after each METH or saline injection. The black arrows indicate the injection times. (**A**) Binge METH induced significant hyperthermia during the treatment. (**B**) During chronic METH regiment, rats developed tolerance to METH-induced hyperthermia. Saline vs. METH: * *p* < 0.05, *** *p* < 0.0001 (two-way ANOVA with repeated measures, followed by the Student-Neuman-Keuls post hoc test). Data are expressed as the mean ± SEM. Abbreviations: METH, methamphetamine; SAL, saline.

Hyperthermia is an important contributing factor in METH neurotoxicity. A decrease in core body temperature decreases METH neurotoxicity, whereas an increase in core body temperature increases toxicity [38,39]. To assess body temperature profiles in both METH regimens, core body temperature was measured prior to the administration of METH or saline and 1 h after each METH or saline injection. As shown if Figure 4A, binge METH administration significantly increased core body temperatures over time. Chronic METH administration initially resulted in a hyperthermic response, which decreased by the seventh day of drug administration (Figure 4B). The chronic METH-induced hyperthermia positively correlated with CpG-2 hypermethylation (Pearson *r*^2^ = 0.99, *p* < 0.01, two-tailed test, *n* = 4); however, group sizes have to be increased to make this result conclusive.

### 3.3. Binge and Chronic METH Differentially Alter the Methylation Status of the ID Element

**Figure 5:**
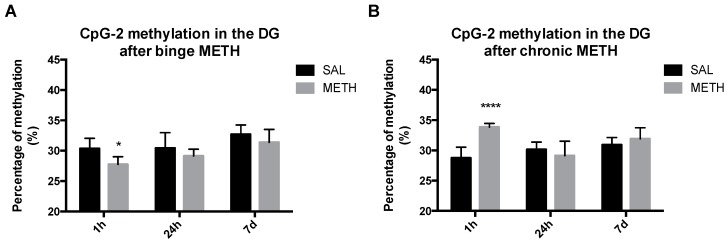
The effects of binge and chronic METH on methylation status of the ID element in the dentate gyrus at 1 h, 24 h and 7 days after drug administration. Adult male Sprague-Dawley rats were administered saline (binge or chronic, 1 mL/kg), binge METH (4 × 10 mg/kg, i.p. every 2 h) or chronic METH (20 mg/kg/day for 10 days, i.p.) and killed 1 h, 24 h or 7 days later. As compared to controls, binge METH triggered significant hypomethylation of CpG-2 site at 1 h (−2.7%, * *p* < 0.05) in the dentate gyrus (**A**), whereas chronic METH triggered significant hypermethylation of CpG-2 at 1 h in the dentate gyrus (5.1%, **** *p* < 0.0001); (**B**). The CpG-2 methylation status returned to the control values within seven days after METH administration. In the binge METH paradigm, there was a significant main effect of time (*F*(2,27) = 7.3, *p* < 0.01) and treatment condition (saline or METH) (*F*(1,27) = 7.6, *p* < 0.05). In the chronic METH paradigm, there was a significant main effect of time (*F*(2,27) = 4.09, *p* < 0.05) and treatment condition (saline or METH) (*F*(1,27) = 8.9, *p* < 0.01), as well as a significant time × treatment interaction (*F*(2,27) = 8.9, *p* < 0.01). Analysis was performed by two-way ANOVA followed by the Fisher LSD post hoc test (*n* = 4–7). Data are expressed as the mean ± SEM. Abbreviations: CpG, C-phosphate-G; DG, dentate gyrus; METH, methamphetamine; SAL, saline.

Retrotransposition of transposable elements does not take place in non-neurogenic areas of adult brain; it can occur in neurogenic niches [6,7,40]. Of note, environmental stressors, including substance abuse, can alter methylation and expression (RNA transcripts) of these elements in non-neurogenic brain areas [7]. To determine whether high-dose binge and chronic METH regimens induce short-term or long-term hypomethylation or hypermethylation of CpG-1 and CpG-2 sites in the dentate gyrus, rats were treated with METH or saline and killed 1 h, 24 h or 7 days after the last injection. As compared to binge saline, binge METH significantly decreased methylation of CpG-2 in the dental gyrus at 1 h after the last METH injection (−2.7%, *p* < 0.05, two-way ANOVA followed by the Fisher LSD post hoc test, *n* = 4–7) (Figure 5A). In contrast to binge METH, chronic METH increased methylation of the CpG-2 site at the 1-h time point (+5.1%, *p* < 0.0001, two-way ANOVA followed by the Fisher LSD post hoc test, *n* = 4–7) (Figure 5B). By Day 7, the methylation of CpG-2 returned to the control values after both METH administrations. There was no change in methylation status of the CpG-1 site at any time point either after binge or chronic METH. Of note, binge METH-treated rats with higher core body temperatures tended to have lower CpG-2 methylation.

### 3.4. PABP1 Protein Levels Are Increased by METH in the Dentate Gyrus

**Figure 6:**
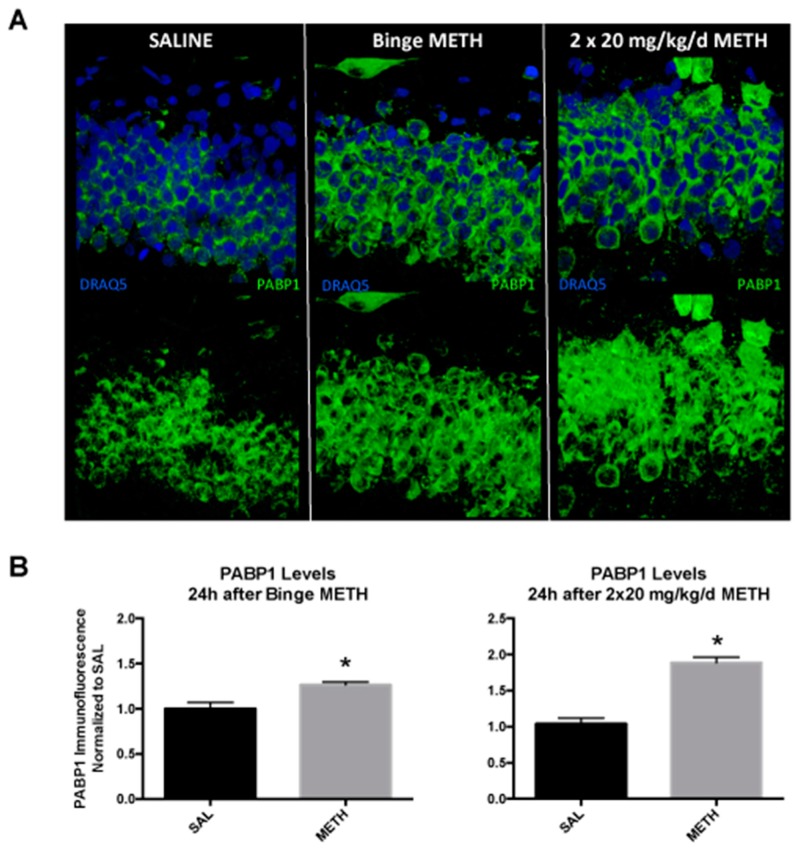
The effects of METH binge and two high doses of daily METH on PABP1 protein levels in the dentate gyrus at 24 h after drug administration. Adult male Sprague-Dawley rats were administered saline (1 mL/kg per injection), binge METH (4 × 10 mg/kg, i.p. every 2 h) or treated with two injections of 20 mg/kg/day METH and killed 24 h after the last injection of saline or METH. (**A**) Compared to saline controls, four doses of 10 mg/kg METH administered every 2 h increased PABP1 immunofluorescence in the dentate gyrus by 26% (*p* < 0.05, Student’s *t*-test, *n* = 4). (**B**) Compared to saline controls, two doses of 20 mg/kg METH administered over a period of two days markedly increased PABP1 immunofluorescence in the dentate gyrus (+88%, *p* < 0.05, Student’s *t*-test, *n* = 4). Data were normalized and expressed as the mean ± SEM. Abbreviations: METH, methamphetamine; SAL, saline; PABP1, poly(A)-binding protein 1.

PABP1 is important for the regulation of mRNA translation and stability, and its levels increase during recovery from a heat shock [41]. As other PABP proteins, PABP1 may bind to SINEs and contribute to the SINE retrotransposition process [13]. To determine whether binge METH-induced ID element hypomethylation at the CpG-2 is accompanied by increased expression of PABP1 in the dentate gyrus, hippocampal slices from rats killed at 24 h after the last dose of binge METH were probed with the anti-PABP1 antibody. Compared to controls, PABP1 immunofluorescence in the dentate gyrus was significantly increased in binge METH-treated rats (+26%, *p* < 0.05, Student’s *t*-test, *n* = 4) (Figure 6A). Based on the methylation data, we hypothesized that tolerance to METH effects developed over the course of its chronic administration. To determine whether PABP1 protein levels are altered in the beginning of the chronic METH regimen, rats were treated with two injections of saline or 20 mg/kg/day METH and killed 24 h after the second injection. Compared to controls, two doses of 20 mg/kg METH administered over a period of two days markedly increased PABP1 immunofluorescence in the dentate gyrus (+88%, *p* < 0.05, Student’s *t*-test, *n* = 4) (Figure 6B).

### 3.5. Chronic METH Induces a Persistent Difference in ID Element Amplification in the Dentate Gyrus

It is known that SINEs can retrotranspose in response to injury [11] and that the ID element can be activated by injury to the CNS [18,19]. However, the hypomethylation of ID element and increase in PABP1 protein levels in the dentate gyrus do not indicate that ID elements retrotransposed upon exposure to binge or chronic METH; these changes only suggest ID element activation. Therefore, we next compared amplification, in other words, the number of copies across the entire genome, of ID element species within gDNA isolated from dentate gyri of the control and chronic METH-treated rats. We detected several loci with significant sequence diversity of clonal ID element populations between the controls and METH-treated samples. The BC1 consensus sequence and ID element type 3 displayed the highest number of reads in the METH and the control samples; however, there was no significant difference between the groups (BC1, SAL: 9303 ± 2026, METH: 8855 ± 764; ID element type 3, SAL: 4763 ± 587, METH: 5120 ± 1060; normalized reads per million; *p* > 0.1, Student’s unpaired two-tailed *t*-test, *n* = 4–5). For ID element type 2b, the detected numbers of reads were: SAL: 598 ± 197; METH: 650 ± 235 (normalized reads per million; *p* > 0.1, Student’s unpaired two-tailed *t*-test, *n* = 4–5). ID element type 1, type 2b and type 2c could not be reliably identified. The ID sequence 29G > A|38G > C|43C > T|52C > T was the sequence that showed the highest, but not significantly different, amplification in the METH group as compared to the control group (six-fold increase, normalized reads per million; SAL: 7 ± 7, METH: 51 ± 22; *p* = 0.14, Student’s unpaired two-tailed *t*-test, *n* = 4–5). Six sequences that could be matched at least once to the reference rat genome were significantly amplified (*p* < 0.05, Student’s unpaired two-tailed *t*-test, *n* = 4–5): 28C > T|38G > C (+61%, SAL: 34 ± 6, METH: 55 ± 2), 29G > A|44G > A|52_53insT (+122%, SAL: 19 ± 6, METH: 42 ± 5), 33G > A|38G > C|39C > G|40A > G (+23%, SAL: 67 ± 4, METH: 82 ± 3), 41A > G (+95%, SAL: 25 ± 9, METH: 49 ± 5), 23delT|29G > A (+162, SAL: 17 ± 4, METH: 45 ± 9) and 28C > G|29G > C|31_32insC|34C > G (+76%, SAL: 30 ± 10, METH: 52 ± 2). Some of these sequences were mapped to the intergenic regions in the rat genome, while some were mapped to genes. Genes associated with preferential amplification and their roles are shown in Table 1. Two ID element sequences that could be matched at least once to the reference rat genome were significantly decreased in the METH group in terms of proportions as compared to the control group: 1G > A|33G > A|39C > G|3T > G|3_4insT and 1G > A|3T > G|3_4insT|43 C> T|52C > T (−55% and −56%, respectively, *p* < 0.05, Student’s unpaired to-tailed *t*-test, *n* = 4–5), suggesting that proliferation of dentate gyrus cells containing these sequences was suppressed by METH.

It is known that SINEs can alter the expression of genes near their insertion site [42]. The precise location of chronic METH-triggered ID element insertions was not possible to determine with the current analysis; however, we detected thirteen ID sequences that did not match even once to the reference genome, but were significantly amplified in the METH group compared to the controls. These ID elements could have been new ID element types or ID element sequences that retrotransposed during chronic METH treatment.

## 4. Discussion

The present study demonstrates that neurotoxic doses of binge and chronic METH regimen differentially affected the CpG methylation within the ID element sequence at 1 h after METH; binge METH triggered hypomethylation, while chronic METH triggered hypermethylation of the CpG-site 2 in the ID element sequence. We also demonstrate that high doses of METH increase the levels of SINE regulatory protein PABP1 in this brain region. Using a new method in NGS analysis, we are the first to demonstrate significant differences in the sequence diversity of clonal ID element populations between controls and chronic METH-exposed samples of dentate gyri. We also identify several ID element sequences that amplified preferentially in certain genomic loci within the reference rat genome.

DNA methylation at the carbon 5 position of the cytosine ring of the CpG nucleotides is the most common epigenetic modification within vertebrate genomes [43,44]. SINEs and LINEs are frequently methylated in mouse, rat and human tissues [44,45,46]. The methylation percentage of CpG sites within the ID element in gDNA from six tissues (liver, lung, kidney, spleen, ovary, testis) from two-month-old male and female Sprague-Dawley rats was found to be about 54% at the CpG-1 and about 60% at the CpG-2 [16]. We found a similar methylation percentage at the CpG-1 and about 30% at the CpG-2 in several brain areas in two-month-old male Sprague-Dawley control rats. The reason for the discrepancy is currently unknown. It might relate to age, tissue specificity and/or methodology. Our data suggest that ID methylation in young adult rats is partial and does not differ between brain regions.

Activation of transposable elements, including SINEs, usually depends on their DNA methylation status and takes place during both embryonic and adult neurogenesis. These elements are silenced by methylation in somatic cells in adults [2,3]. Activation of transposable elements is observed in several diseases and can be triggered by environmental stimuli. Thus, hypomethylation and increased expression of SINEs was reported in cancer, autoimmune and neurodegenerative diseases, substance abuse [1,3,7] and upon exposure to several cellular stressors, including thermal stress and hypoxia [47,48,49,50]. An acute low dose of METH (4 mg/kg) was shown to decrease the expression of DNA methyltransferase 2 in the dentate gyrus of Wistar rats when measured 24 h later [51]. This finding and our finding of ID element hypomethylation at 24 h after the high-dose binge METH administration suggest that both low and high METH doses might be able to trigger hypomethylation of the element. The extent of ID element hypomethylation at the CpG-2 site was small (2.7%). Similarly, less than 10% hypomethylation of *Alu* was detected in cancer cells [52,53] and Klinefelter syndrome [54]. Less than 10% hypomethylation of the B1/B2 element was found after exposure to soil dust and traffic exhaust [55]. As in our experiment, the weak increase in mouse SINEs, detected at the 24-h time-point, was followed by their hypermethylation later on [55]. Activation of transposable elements is more complex than simultaneous hypomethylation of all CpG sites [2,50,56]. This fact could explain, in part, the lack of changes in the methylation state at the CpG-1 site.

At 24 h after chronic METH, the CpG-2 site of the ID element was hypermethylated and returned to the control levels by the seventh day of withdrawal from METH, suggesting that the ID element was silenced by de novo methylation sometime during chronic METH administration. This conclusion is supported by other published studies that observed increases in global DNA methylation after chronic administration of low doses of amphetamines. Thus, in adult rats, chronic exposure to amphetamine (1 mg/kg) led to increases in global DNA methylation in the nucleus accumbens, prefrontal cortex and olfactory tubercle [57], while chronic exposure to METH (4 mg/kg or 0.5–3 mg/kg) resulted in increased expression of DMT1 (DNA-methylating enzyme 1) in the striatum and nucleus accumbens [58,59] at two weeks after drug administration.

It has been proposed that methylation of histones rather than methylation of gDNA plays a dominant role in transcription of SINEs [56]. Therefore, it is possible that the hypermethylation of the ID element did not suppress its activity, but histone methylation did. Along these lines, neurotoxic self-administered METH increased the levels of histone-3 methylation in the striatum at 1 h and 24 h after cessation of METH administration [60]. Similarly, hypomethylation of CpG-2 might have played a small or no role in ID element activation. Of note, binge METH-treated rats with higher core body temperatures tended to have lower CpG-2 methylation, which together with the data on increased expression of SINEs from several different species following thermal shock [47,48,49] suggest that binge METH-induced hyperthermia leads to hypomethylation of the ID element. In addition to thermal shock, ID element activation and increased expression can be induced by inflammatory response [61], inhibition of protein synthesis [48] or DNA breakage, with the latter being known to also induce SINE retrotransposition [11]. Consequently, the increased expression of the ID element in the dentate gyrus may have occurred not only due to the thermal shock, but also due to METH-induced inflammatory response [62], DNA breakage [63,64,65] and/or inhibition of protein synthesis [66], with at least some of these elements retrotransposing within gDNA.

PABPs are multifunctional RNA-binding proteins that regulate multiple aspects of mRNA translation and stability [67], including mediating retrotransposition of SINEs. SINEs are non-autonomous elements [8]. They use PABPs and proteins encoded by endogenous LINE-1s [4,9,10,11] to form RNP complexes that are needed for the transport of SINEs to the nucleus for reverse transcription [42]. PABP1 is a protein suggested to positively regulate reverse transcription of the ID element-containing BC1 gene [13] and LINE-1 [68], as well as to have a role in cellular recovery from thermal stress [41]. Consequently, the increase in PAB1 levels observed by us at 24 h after binge METH and on the third day of chronic METH treatment could have mediated either stabilization of ID element RNA and ID element retrotransposition, or re-initiation of protein synthesis after METH-induced thermal shock, or both. The more pronounced increase in PABP1 levels in the dentate gyrus after two daily doses of 20 mg/kg METH than after binge METH (4 × 10 mg/kg, every 2 h) could have been due to the difference in the timeline, i.e., more PABP1 protein was synthesized over three days than one day since the first METH injection.

ID element amplification within gDNA was detected after METH treatment, which is the most important finding of our study. The most abundant subtypes of ID element (e.g., types 3, 4, etc.) were detected in our analyses; however, they were not significantly different between the groups, suggesting that the significantly amplified sequences originated (copied) from different locations than these sequences’ loci within the rat genome [69,70] and that BC1, ID element type 2b, type 3 and type 4 activities were suppressed during METH administration.

We identified several subtypes of ID elements, closely related to the master BC1 sequence, that were significantly affected by METH treatment. Certain subtypes, like 28C > T|38G > C, were associated with a broader set of genes compared to other subtypes, such as 29G > A|44G > A|52_53insT. This may indicate that the distribution of ID element subtypes could be substantially different and that this further may be influenced by METH treatment, although further work is needed to test these hypotheses. Although not described in Table 1, ID element insertion in the same gene at multiple locations was also present with several subtypes. The reason for this apparent targeting of certain genes by ID element subtypes is unknown, but could suggest the possible importance of a gene or even non-specific effects by METH. In addition to ID element subtypes being found within genes, many mapped locations were in intergenic locations not associated with a particular gene. Intergenic transposable elements could be important when considering genomic stability and nearby gene regulation. One explanation for the lack of increases in the number of BC1 sequences and increases in other ID elements is the fact that the older subfamilies of this element appear to be incapable of retrotransposition, with younger families being capable of amplifying [71] from numerous source genes [70]. In agreement with the latter finding, we identified multiple potential gene sources of the ID element in the rat dentate gyrus. Secondly, different cell populations within the rat dentate gyrus might have reacted differently to METH [22]. Thirdly, duplications and deletions of the element may have taken place [72].

Of the most significant ID element subtypes that were preferentially amplified in the METH group, the associated genomic locations had a diverse range of functions. For example, ID element subtype 28C > T|38G > C was found in genes coding for transporters (*SLC12A2*, *SLC9A9*), protein modification (*UBA6*, *Map3k12*) and transcriptional regulation (*CIPC*). Others, such as *GPC5* [73,74], have been implicated in addiction, while *UBA6* hypomethylation has been found with METH treatment [75]. Overall, the genes associated with the identified ID element subtypes require further, targeted investigation to understand what effects METH has on their expression levels. The current investigation was limited by a low power to detect statistical significance and the ability to see absolute differences, making future work in this area important. We believe the novel approach to identify ID element changes with METH treatment overcomes these limitations and has identified important effects that can be further investigated.

## 5. Strengths and Limitations

Strengths of the current work include a novel and unbiased approach to characterizing ID element changes in the brain. Additionally, our work assessed both pre- and post-translational changes (e.g., epigenetic to protein expression). The limitations of our study are relatively small sample sizes, the inability to detect all ID element subtypes and the pilot character of the study due to the primer design (the primers were designed to be contained within the consensus sequence in order to improve NGS performance). Finally, the lack of a targeted qPCR and sequencing analysis of ID elements makes comparisons to other studies difficult. Nevertheless, this work provides candidate genes and locations from which our identified ID element subtypes may have originated in the reference genome. We plan to improve and validate the method in the near future through replication and a better understanding of the destination of ID element retrotransposition.

## 6. Conclusions

In summary, our results suggest that high-dose chronic METH triggers a chain of events leading to ID element retrotransposition in the dentate gyrus and that the ID element may play a role in METH-mediated changes in hippocampal neurogenesis. The hippocampus is involved in the relapse effect of drug abuse and memory tasks commonly impaired in human chronic METH users [23,76,77]. Consequently, determination of METH effects on the ID element and other SINEs is clinically relevant to abuse of amphetamines and the related decline of cognitive skills. Identification of ID-containing genes affected by high METH doses adds to knowledge on SINE-related epigenetic events induced by neurotoxic doses of binge and chronic METH. Further work will aid in understanding the origin of the ID elements that change their position in the genome after chronic METH treatment.

## Figures and Tables

**Table 1:** Top six preferentially amplified ID element subtypes and their associated genomic locations. Dorsal dentate gyrus samples were analyzed using next-generation sequencing technology to identify ID element subtypes that were preferentially amplified in rats administered methamphetamine (METH) relative to rats administered saline.

ID Element Subtype ^a^	% Change ^b^	*p*-Value	Genes Associated with Preferential Amplification ^c^
28C > T|38G > C	61	0.00828	Cipc (Chr6:110986686), Slc9a9 (Chr8:102453600), LOC680227 (ChrX:74311735), Ift80 (Chr2:165544369), Mfsd14b (Chr17:2643624), Slc12a2 (Chr18:52921730), Uba6 (Chr14:23511767), Lasp1 (Chr10:85756102), Dcakd (Chr10:91116391), Smpdl3b (Chr5:150930976), Atp2b2 (Chr4:145726282), Smad9 (Chr2:144054261), Asic2 (Chr10:68890335), Abca17 (Chr10:13680892), Rab3c (Chr2:41772443), Ralgps2 (Chr13:74477445), Gtf2e2 (Chr16:62133089), Map3k12 (Chr7:144103811)
29G > A|44G > A|52_53insT	122	0.01785	Veph1 (Chr2:157964489), Mapk8ip3 (Chr10:14288892)
33G > A|38G > C|39C > G|40A > G	23	0.01951	Proximal to Cep162 (Chr8:94937230)
41A > G	95	0.03976	Hdac4 (Chr9:99135435), Atg3 (Chr11:60587858), Nutf2 (Chr19:37841169), Urm1 (Chr3:8399609), Rsl1d1 (Chr10:4498654), Kpna3 (Chr15:41704423), Cnga3 (Chr9:43837574), Tfap2d (Chr9:25355932), Crkl (Chr11:87792847), Atp10d (Chr14:38491114), Vegfd (ChrX:31818688), Slc24a3 (Chr3:139780502), Pbx1 (Chr13:86476988), Prpsap1 (Chr10:105447151), Mylk (Chr11:69140146), Synpo Chr18:55910398), Zbtb46 (Chr3:176916873), Rtcd1 (Chr2:219556431), Slit3 (Chr10:20429924), Lcmt1 (Chr1:193375030), Rab30 (Chr1:157633572), Sepw1 (Chr1:77806751), Tjap1 (Chr9:17089159), Laptm4b (Chr7:72935301), Optn (Chr17:77215077), Nup210l (Chr2:189478449), Aaas (Chr7:143939751), Astn2 (Chr5:82045057), Rb1cc1 (Chr5:13068263), Smarcal1 (Chr9:79976755), Gsto2 (Chr1:267621506), Sppl3 (Chr12:47334193), Tctn2 (Chr12:37366571), Impdh1 (Chr4:56485763), Tbc1d14 (Chr14:79314924), Tbc1d8 (Chr9:46159589), Fcgr2b (Chr13:89356250), Scarb1 (Chr12:36712849), Rab3ip (Chr7:59939610), Prpf40a (Chr3:38695276), Utp20 (Chr7:29314493), Arsb (Chr2:23413525), Entpd7 (Chr1:263467744), Capn2 (Chr13:100899435), Txndc11 (Chr10:4613068), Gspt1 (Chr10:4386117), Six4 (Chr6:95989936), Susd5 (Chr8:122359614), Il23r (Chr4:98243720), Casc4 (Chr3:113853687), Ranbp17 (Chr10:18194793), Gabrb1 (Chr14:38814344), Cdc37 (Chr8:22163945), Fam172a (Chr2:5339706), Gtf2f2 (Chr15:57997967), Adra1a Chr15:43354439), Pum2 (Chr6:33799082), Gpr137b (Chr17:90744301), Atp1b3 (Chr8:104217176), Ubap1 (Chr5:57741520), Sgpl1 (Chr20:30724101), Ptprk (Chr1:17762449), Wwp2 (Chr19:39600430), Sbno1 (Chr12:37628774), Zfand3 (Chr20:8810745), Wrn (Chr16:62554494), Kyat3 (Chr2:248661110), Aldh2 (Chr12:40485627), Dpp6 (Chr4:4219884), Plekho2 (Chr8:71098823)
23delT|29G > A	162	0.04303	Taar8c (Chr1:22334448)
28C > G|29G > C|31_32insC|34C > G	76	0.04654	Ythdf2 (Chr5:150375359)

^a^ The reference sequence on which ID element subtypes were aligned and identified was the BC1 consensus sequence: GGTTGGGGATTTAGCTCAGTGGTAGAGCGCTTGCCTAGCAAGCGCAAGGCCCTGGGTTCGGTCCTCA. Each subtype name is represented by the changes it contains compared to the reference sequence. For example, for the ID element subtype, 28C > T|38G > C; this indicates that the 28th and 38th positions with respect to the consensus sequence have been changed to T and C, respectively. #_#ins indicates an insertion of a nucleotide between the given positions. #del indicates the nucleotide position that was deleted. ^b^ METH relative to controls. ^c^ Gene regions to which each ID element subtype mapped. Intergenic regions are not shown. Genomic coordinates using Rat Genome Sequencing Consortium (RGSC) 6.0/rn6.

## Abbreviations

The following abbreviations are used in this manuscript:

DMT	DNA methyltransferase
ID element	Identifier element
LINEs	Long interspersed elements
METH	Methamphetamine
PABP	Poly(A)-binding protein
SAL	Saline
SINEs	Short interspersed elements
